# Synergetic Effect of Organic Flocculant and Montmorillonite Clay on the Removal of Nano-CuO by Coagulation-Flocculation-Sedimentation Process

**DOI:** 10.3390/nano11102753

**Published:** 2021-10-17

**Authors:** Rizwan Khan, Muhammad Ali Inam, Kang-Hoon Lee, Abdul Sami Channa, Mukhtiar Ali Mallah, Young-Min Wie, Mahmood Nabi Abbasi

**Affiliations:** 1 Department of Chemical Engineering, Quaid-e-Awam University of Engineering, Science and Technology (QUEST), Nawabshah 67480, Pakistan; rizwansoomro@quest.edu.pk (R.K.); abdul.sami@quest.edu.pk (A.S.C.); mukhtiarmallah@quest.edu.pk (M.A.M.); mahmoodabbasi73@quest.edu.pk (M.N.A.); 2 Institute of Environmental Sciences and Engineering (IESE), School of Civil and Environmental Engineering (SCEE), H-12 Campus, National University of Sciences and Technology (NUST), Islamabad 44000, Pakistan; ainam@iese.nust.edu.pk; 3 Department of Civil and Environmental Engineering, Hanyang University, 222 Seongdong-gu, Seoul 04763, Korea; 4 Department of Materials Engineering, Kyonggi University, Suwon 16227, Korea; supreme98@kyonggi.ac.kr

**Keywords:** Box–Behnken design, coagulation-flocculation-sedimentation, clay, optimization, CuO

## Abstract

The widespread usage of nano-copper oxide particles (nano-CuO) in several industrial products and applications raises concerns about their release into water bodies. Thus, their elimination from drinking water is essential to reduce the risk to human health. This work investigated the removal of nano-CuO from pure water and montmorillonite clay (MC) suspensions using poly aluminum ferric chloride (PAFC) as well as cationic polyacrylamide (PAM) by the coagulation-flocculation-sedimentation (C/F/S) process. Moreover, the PAFC and PAFC/PAM flocculation performance for various nano-CuO particles concentrations, dosages, pH, settling times and stirring speeds were also investigated. The findings showed that the removal of nano-CuO and turbidity in MC suspension were higher as compared to pure water. Moreover, the combined effect of PAFC/PAM on the elimination of nano-CuO and turbidity was also substantially better than the individual use of PAFC or PAM. The efficient removal of CuO was observed in the solution containing higher mass concentration in the order (10 mg/L > 2.5 mg/L > 1 mg/L) with an increased coagulant dose. The improved removal performance of nano-CuO was observed in a pH range of 7–11 under various water matrices. The C/F/S conditions of nano-CuO were further optimized by the Box–Behnken statistical experiment design and response surface methodology. The PAFC/PAM dose resulted in the maximum removal of nano-CuO (10 mg/L) in both pure water (>97%) and MC suspension (>99%). The results of particle monitoring and Fourier transform infrared of composite flocs revealed that the main removal mechanism of nano-CuO may be the combined effect of neutralization, complexation as well as adsorption.

## 1. Introduction

Developments in nanotechnology have led to the widespread application of several metal-based nanoparticles (NPs) in different products and processes. The increased usage of NPs results in their release into the ecosystem, thereby affecting aquatic life and human health via the food chain [1]. Metal particles such as nano-copper oxide (nano-CuO) are typical metal-based particles, with an estimated global production of around 570 tons/year, which is projected to enhance over time [2,3]. The failure of wastewater treatment processes to effectively remove large quantities of nano-CuO can lead to the contamination of freshwater sources. The hazardous effects of nano-CuO upon many biological species, for instance, Lymphocytes, Fagopyrum esculentum, Daphnia magna and Pseudokirchneriella are well known [4,5,6,7,8]. Moreover, human exposure to these metal-based NPs via ingestion may cause cytotoxicity as well as genotoxicity even at a low concentration of 1 µg/mL [1,9]. Consequently, failure to remove nano-CuO effectively might increase the potential risk of exposure of these NPs to aquatic life and humans.

The mobility of metal-based NPs in the aquatic environment is controlled by different technologies such as bioremediation, phytoremediation, ion exchange, membrane separation, adsorption, electrochemical and coagulation processes. In wastewater treatment plants, biological treatments such as activated sludge can remove nano-CuO particles. However, the toxicity of NPs on the bacterial film of activated sludge interferes with the overall removal process by modifying the properties of sludge [10,11]. The membrane process can also effectively remove the NPs from water, but it is not commercially viable due to membrane fouling caused by highly concentrated NPs [12,13]. The incomplete removal of NPs through sewage treatment may eventually increase the threat of freshwater contamination. Consequently, a cost-effective method for complete nano-CuO particle removal is imperative. 

Previous studies have confirmed that the conventional coagulation-flocculation-sedimentation (C/F/S) method can be used for the effective removal of various metal-based NPs such as nano-CuO, TiO_2_, cadmium telluride (CdTe), multiwall carbon nanotubes (MWCNT) and zinc oxide (ZnO) from water. In alum treated water, Chalew et al. reported residual concentrations of 48–99%, 3–8% and 2–20% in spiked ZnO, TiO_2_ and AgO NPs, respectively [14]. Earlier reports suggested that ferric chloride (FeCl_3_) yielded a better coagulation efficiency of TiO_2_ and ZnO NPs under a heterogeneous environment compared to polyferrous sulfate (PFS) and polyaluminum chloride (PACl) [15]. Moreover, the removal of metal-based NPs might be enhanced by the higher dosages of FeCl_3_ and Al_2_(SO_4_)_3_ [16,17]. The C/F/S process appears effective in removing metal-based NPs; however, the higher coagulant dose and unstable performance might be related to the single aluminum-based or iron-based coagulant. A recent study [18] described that the combined effect of aluminum and iron salt coagulants such as polyaluminum ferric chloride (PAFC) enhanced the coagulation performance of TiO_2_ NPs at a low dosage. Moreover, high-molecular-weight flocculants such as cationic polyacrylamide (PAM) were reported to enhance the impact of removal via charge neutralization of the negatively charged flocculants [19,20]. Thus, a comprehensive exploration of the concurrent effect of inorganic coagulant and organic flocculants is essential for the removal of nano-CuO particles from aquatic environments. 

Earlier studies focused on the C/F/S behavior of the primary pollutant nano-CuO; however, the interaction between several substances hindered the aggregation and removal of NPs. The interaction between clay particles such as kaolinite and NPs enhances the aggregation in ground and surface water, although the special structure of clay significantly affects the coagulation performance of NPs [21]. Wang et al. demonstrated the effect of anisotropy aggregation on the stability of NPs and clay mineral mixtures [22]. Other researchers have also shown the high adsorption capacity of the modified clay minerals of heavy metal ions under coagulation conditions [6]. Consequently, it is essential to systematically analyze the effect of clay minerals on the fate, mobility and removal behavior of nano-CuO during the conventional C/F/S process. 

Many researchers have used the classical approach known as the single-factor method to investigate the coagulation behavior, although this technique fails to predict the interactive behavior under different operating parameters [23]. Amongst many statistical techniques, the Box–Behnken statistical experiment design (BBD) is a classical response surface methodology (RSM) used for modeling and analysis of experimental data with multiple operating parameters [24]. To the best of our knowledge, studies that simulate the C/F/S performance of nano-CuO removal using RSM have not been reported yet. It is crucial to systematically investigate the removal performance of nano-CuO by the C/F/S process using a mathematical modeling approach.

The present study aims to investigate the coagulation behavior of nano-CuO particles in water using PAFC and PAM via C/F/S under different experimental conditions. The effects of various mass concentrations of nano-CuO in pure water and clay suspensions were investigated. First, the optimum coagulation conditions of nano-CuO NPs were explored under PAFC, PAM and PAFC/PAM using a one factor at a time approach. Secondly, BBD and RSM were used to investigate the effect of various factors such as inorganic coagulant, organic flocculant and stirring speed. Last, the validity and reliability of the statistical analysis with various experimental data points were determined by comparing experimental and predicted nano-CuO removal efficiency response values. 

## 2. Materials and Methods

### 2.1. Materials 

Nano-copper oxide powder with an average <50 nm diameter, 99.8% purity, and montmorillonite clay (MC) used as the clay minerals in the current study were obtained from Sigma-Aldrich (St. Louis, MO, USA). The inorganic coagulant polyaluminum iron chloride (PAFC) (containing 26% of Al and Fe) and organic flocculant cationic polyacrylamide (PAM) with a molecular weight of 12 million were obtained from water treatment material Gongyi Tenglong Co., Ltd., Henan, China and Tianjin Chemical Reagent, Tianjin, China, respectively.

### 2.2. Stock Solutions

The nano-CuO stock solution was prepared by mixing various concentrations of CuO powder in pure water (18.2 MΩ). Initially, the stock solution of 100 mg/L of CuO was prepared and the final pH of the stock suspension was adjusted to 7.0 using 100 mM NaOH or HCl. The NP solution was dispersed by a probe-type ultrasonicator (Bio-safer 1200-90, Nanjing, China) for a period of 30 min at 400 W to acquire a well stable nano-CuO particles suspension. The 1 g/L MC suspension was prepared by weighing 1 g of MC powder using the microbalance (Mettler Toledo AG, Ag Model-XP26DR, Greidensee, Switzerland) and dissolved in 1 L pure water. Before the experiment, NPs’ suspension was dispersed by a probe-type sonicator for 30 min. The stock solution comprising PAM 100 mg/L was prepared in pure water. The solution was stirred at a temperature of 50 °C and 250 rpm for 1 h with a lab magnetic stirrer to ensure complete dissolution. The dry weight method was used to calculate the dosage of inorganic coagulant (PAFC) and organic flocculant (PAM) in the current C/F/S experiments.

### 2.3. C/F/S Experiments

The experiments were conducted in the jar tester with a six-synchronous automatic lifting mixer (Young Tech Co., Ltd. Gyeongsangbuk-Do, Korea). Pure and artificial test water (MC suspension) were transferred to glass beakers. Predetermined amounts of PAFC, PAM and PAFC/PAM were dosed, and the suspension was rapidly stirred for 2 min at 250 rpm, and slow stirred for 10 min at 70 rpm. The different sets of the experiment including nano-CuO concentration (1–10 mg/L), pH (6–11), settling time (5–30 min) and stirring speed (100–350 rpm) were also investigated. The samples were collected 2 cm below the solution in the sampling vessel after the completion of the experiment for further analysis. The particle size was analyzed through the particle-size analyzer Zetasizer (Nano ZS90, Worcestershire, UK). During the coagulation, the water sample containing flocs was circulated using a silicone tube (dia 8 mm) through a peristaltic pump with controlled flow rate into the Zetasizer.

### 2.4. Analytical Procedure

Turbidity removal was determined with a turbidity measurement of the supernatant using a turbidimeter (Hach 2100-N, Loveland, CO, USA). Moreover, the residual concentration of nano-CuO was calculated by determining the absorbance at 254 nm with a UV-Vis spectrophotometer (Optizen, Mecasys, Daejeon, South Korea) as shown in the supplementary information (Figure S1). Additionally, a Fourier transform infrared (FTIR- JASCO, Easton, PA, USA) in the range of 400 to 4000 cm^−1^ was performed before and after the C/F/S experiments to understand possible removal mechanisms. Each test was performed three times and the relative standard deviations (STD) were reported as <5%. Moreover, the standard error was calculated through the division of STD by the square root of the number of tests performed. The popular Design Expert (version 8.0.5) software was used for the regression analysis, response-surface and contour maps of the experimental data.

## 3. Results

### 3.1. Influence of Coagulant and Flocculant Dosage on the Removal of Nano-CuO

**Figure 1:**
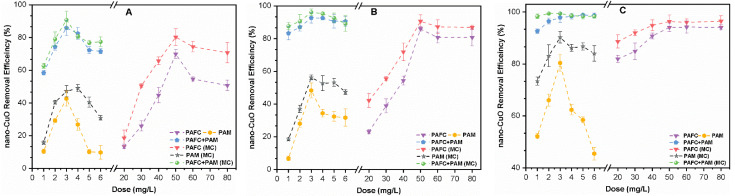

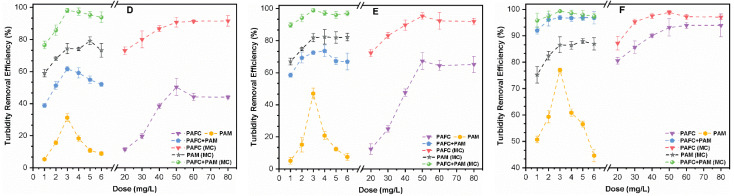
Influence of coagulant and flocculant dosage on the removal efficiency of nano-CuO and turbidity for various mass concentrations (**A**,**D**) 1 mg/L, (**B**,**E**) 2.5 mg/L and (**C**,**F**) 10 mg/L, respectively.

Figure 1 shows the influence of PAFC, PAM, and PAFC/PAM on the removal performance in two water environments with varying concentrations (i.e., 1, 2.5 and 10 mg/L) of nano-CuO. The optimum dose of PAM (3 mg/L) and PAFC (50 mg/L) was observed for removing nano-CuO from pure water as well as MC containing water. Increasing the dosage of PAFC and PAFC/PAM led to the enhanced removal rate of nano-CuO and turbidity as depicted in Figure 1. The excess dose of PAM significantly decreased the removal rate after the optimal critical point; however, this effect of removal was found insignificant in the case of PAFC/PAM. It was observed that the removal efficiency of nano-CuO was enhanced with increasing mass concentration in the solution. This might be related to the fact that the higher concentration of NPs enhanced the probability of collision as well as increased the effect of co-precipitation via formation of the floc core. These findings are in good agreement with earlier work by Honda et al. [15]. The relatively better removal rate of various nano-CuO (1, 2.5 and 10 mg/L) concentrations in pure water was observed in PAFC/PAM (83.33%, 92.78% and 98.54%) rather than PAFC (72.48%, 86.23% and 93.90%) alone. The efficacy of co-precipitation and adsorption among the flocculants as well as NPs improved with the addition of PAFC/PAM in the solution. The enhanced removal efficiency of nano-CuO was found in suspension containing MC. Moreover, the addition of PAFC in both the pure and MC environments increased the removal rate of nano-CuO (10 mg/L) from 94.60% to 97.34%. As illustrated in Figure 1, the addition of MC and PAM had beneficial effects on the nano-CuO removal rates and the turbidity of the solution with similar flocculation conditions. In addition, the combined effect of PAFC/PAM improved the nano-CuO removal compared with PAFC as shown in Figure 1.

In pure water, the single system of nano-CuO (10 mg/L), the removal efficiency of PAFC before and after the addition of PAM was 94.90% and 98.54%, respectively. The PAM resulted in the formation of larger stable flocs due to increased adsorption bridging of the linear polymer, thereby improving the flocculation of nano-CuO [25]. The turbidity removal efficiency was strongly correlated with the removal of nano-CuO. It was observed that after optimal dosage, the increase of the PAM dosage decreased the rate of turbidity removal by 38%; however, this effect was found to be insignificant in the PAFC and PAFC/PAM cases. These results can be attributed to the fact that PAFC has strong potency to ionize high amounts of Fe and Al cations in the aquatic environment; thereby neutralizing the negative surface potential of nano-CuO [26]. However, the residual cations enhanced the effect of steric hindrance among colloids, resulting in particle stabilization. For the MC system, the removal efficiency was slightly higher compared with CuO in pure water. This phenomenon may be related to the fact that the addition of MC increased the floc nucleus and collision probability of the NPs, thereby resulting in adsorption onto the flocculant surface and formation of tiny dense flocs [27]. During flocculation, the simultaneous effects of PAFC and MC provided a net sweep, thus forming compact flocs and improving the overall effect of flocculation and impeded cohesion.

### 3.2. Influence of pH on the Removal of Nano-CuO

**Figure 2:**
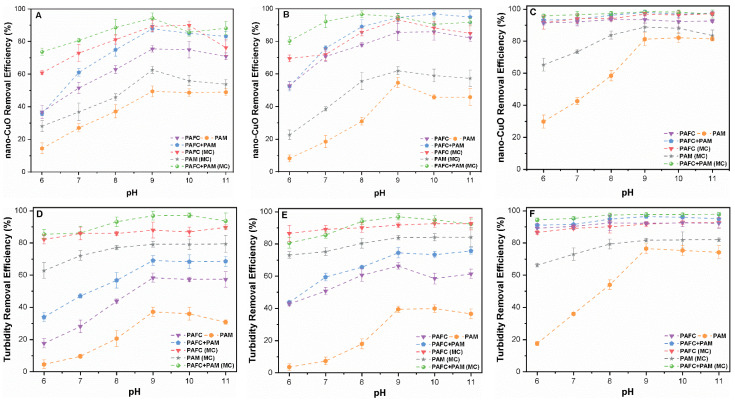
Influence of pH (6–11) on the removal efficiency of nano-CuO and turbidity for various mass concentrations (**A**,**D**) 1 mg/L, (**B**,**E**) 2.5 mg/L and (**C**,**F**) 10 mg/L.

Figure 2 shows the effect of pH on the removal rates of nano-CuO and turbidity within the pH range of 6–11 at optimum doses of PAFC (50 mg/L) and PAM (3 mg/L). Fluctuation of the pH in the two systems significantly affected the removal of nano-CuO and turbidity. For instance, the removal efficiency was remarkably enhanced with the increase in pH and then declined to different levels. In the absence of MC suspension, PAM resulted in less than 30% removal of nano-CuO and turbidity at pH 6. The addition of MC enhanced the removal rates of nano-CuO and turbidity up to 60% under similar flocculation conditions as shown in Figure 2. However, the system containing MC had an insignificant effect on the nano-CuO removal by PAFC and PAFC/PAM. At pH 9 the removal efficiency of various initial concentrations of nano-CuO enhanced to the maximum level. In the MC environment, the removal efficiency of nano-CuO by PAFC/PAM was found to be more than 90%. In general, under acidic pH, the removal of both nano-CuO and turbidity was reduced compared with slightly alkaline environments and this difference was significant in the pure water system. The removal efficiency of PAFC, PAM and PAFC/PAM in the alkaline condition remained the same, suggesting that the higher pH environment had a negligible effect. The results of nano-CuO and turbidity removal curves showed similar trends, which indicate a strong correlation between the initial mass concentration of nano-CuO and turbidity. Our observation is consistent with earlier findings [28].

In the aquatic environment, the pH plays an important role in the surface potential of nano-CuO and also affects the formation of flocculant hydrolysates. The removal efficiency of PAM was mainly influenced by the solution pH due to a positive charge at acidic and alkaline conditions. Under the acidic environment, the hydroxyl groups on the surface of nano-CuO adsorbed protons and thus were positively charged, while PAM released cations in the solution, resulting in the poor removal efficiency of NPs. The surface potential of CuO above pH 7 was negatively charged owing to the loss of protons. The release of cations due to the ionization of PAM neutralized the surface charge thereby enhancing the removal due to formation of large aggregated flocs in the system [29]. However, under highly alkaline pH environments, both NPs and PAM were negatively charged thus increasing the electrostatic repulsion amongst them, and so reducing the overall flocculation and precipitation efficiency. Furthermore, under alkaline conditions, PAFC rapidly hydrolyzed and precipitated to form hydroxide, thereby reducing the overall removal efficiency of NPs. The effect of nano-CuO destabilization was enhanced by increasing the solution pH following the addition of PAFC; however, the tiny flocs remained suspended in the solution [30]. The compounding process of PAFC/PAM was relatively more stable than PAFC and PAM alone due to the wider pH adaptation range of organic flocculant (PAM) [31,32]. Moreover, the complex structure of the PAFC/PAM polymer remarkably enhanced the flocculant-specific surface area and improved the bridging effect during adsorption [32]. Consequently, the combination of inorganic and organic flocculants had a synergistic effect on the C/F/S process and on the overall nano-CuO removal performance from the aquatic environment.

### 3.3. Influence of Sedimentation Period on the Removal of Nano-CuO

**Figure 3:**
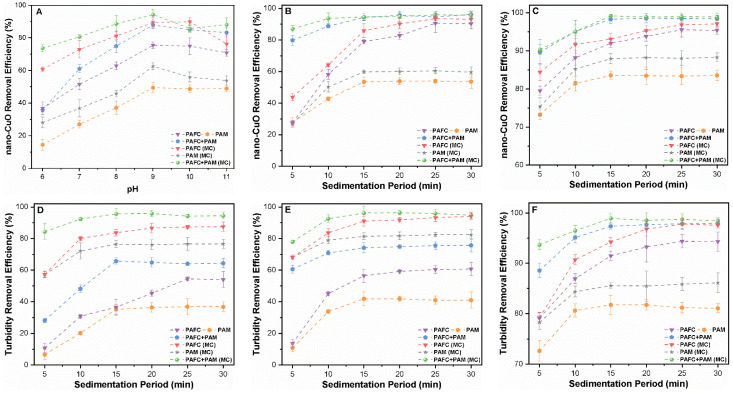
Influence of settling time (5–30 min) on the removal efficiency of nano-CuO and turbidity for various mass concentrations (**A**,**D**) 1 mg/L, (**B**,**E**) 2.5 mg/L and (**C**,**F**) 10 mg/L.

The sedimentation period, which is directly related to the design of the settling tank, is considered as a key parameter affecting the magnitude of design, investment and operation cost. In the present study, the settling time was used as the analytical index based on the optimal dosage of coagulant. The removal rates of nano-CuO and turbidity under specific PAFC, PAM and PAFC/PAM settling time were investigated with various concentrations of nano-CuO (1, 2.5 and 10 mg/L) in two separate systems. Figure 3 shows the effect of the prolonged duration of precipitation in increasing the turbidity and removal efficacy of nano-CuO. The addition of the PAFC coagulant resulted in stable removal rates of CuO after 30 min of sedimentation time in both systems. In the single CuO system, the removal performance of PAFC and PAM for 1, 2.5 and 10 mg/L CuO under a settling time of 5 min reached 23.38, 26.04 and 79.13% and 13.87, 26.10 and 73.11%, respectively. However, the removal rates remained constant after 15 min of settling time. The precipitation period under different concentrations of CuO was substantially shorter in the composite coagulant-flocculant system (PAFC/PAM) compared to the single PAFC and PAM. The removal efficiency of nano-CuO by PAFC/PAM exceeded 75% within 5 min of settling. In the single system of CuO, the removal rate of 1, 2.5 and 10 mg/L CuO by PAFC after 5 min of settling increased from 23.38% to 65.17%, 26.04% to 78.54% and 79.13% to 92.94%, respectively. In general, the nano-CuO removal rates and turbidity stabilized with a further increase in the precipitation period up to 20 min. The optimal removal rate of nano-CuO (10 mg/L) after 30 min by PAFC, PAM and PAFC/PAM was found to be 94.14%, 83.89% and 99.24%, respectively. Moreover, the addition of MC facilitated the removal efficiency of CuO and turbidity under similar settling conditions as depicted in Figure 3. The removal efficiencies, turbidity and settling time of nano-CuO in the three initial mass concentrations were similar in both systems. Our results are consistent with those reported in the literature [33].

In the PAFC case, the optimal settling period of CuO was found to be shorter than that of PAM alone. The ionization of PAFC released Fe(III) and Al(III) ions in the solution, leading to the formation of complexes with nano-CuO. Moreover, PAM contained the reactive groups in its polymer chains which provided the favorable adsorption sites for tiny flocs of CuO. Thus, the removal of nano-CuO by PAM mainly occurs via the adsorption bridging mechanism [34]. These observations are consistent with the literature [35], which reported similar turbidity removal rates of the kaolinite−humic acid solution using PAFC and PAM−PAFC. In the PAFC/PAM case, stable nano-CuO (10 mg/L) and turbidity removal rates of up to 97.85% and 96.45%, respectively, were observed after 15 min as shown in Figure 3C,F. The enhanced removal efficiency may be related to the bridging effect of PAFC/PAM, which forms dense and stable flocs that rapidly settle down. In contrast, the flocs formed by PAFC and PAM alone were small and had longer settling times. Thus, the combination of organic and inorganic flocculants, i.e., PAFC/PAM can significantly decrease the treatment cost by reducing the settling time. 

### 3.4. Influence of Stirring Speed on the Removal of Nano-CuO

**Figure 4:**
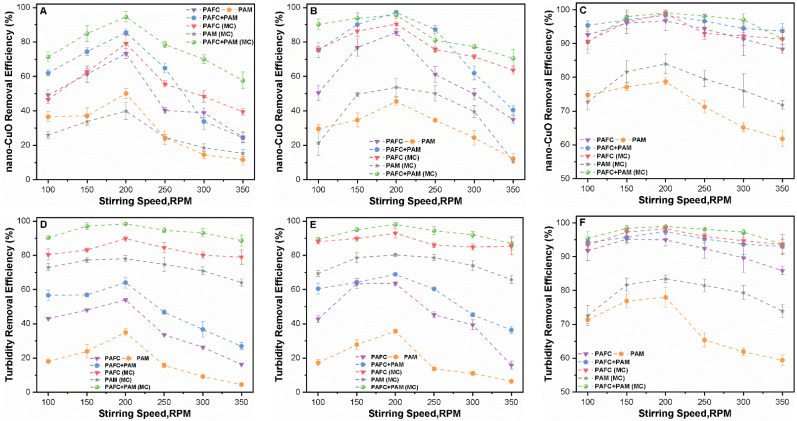
Influence of stirring speed (100–350 RPM) on the removal performance of nano-CuO and turbidity for various initial mass concentrations (**A**,**D**) 1 mg/L, (**B**,**E**) 2.5 mg/L and (**C**,**F**) 10 mg/L.

The optimum doses of PAFC and PAM were determined at neutral pH and a precipitation duration of 30 min, at 10 min intervals under a slow stirring speed of 70 rpm and at 2 min under a fast rotation speed (FRS) of 250 rpm. Figure 4 shows the effect of different stirring speeds on the elimination of nano-CuO particles. The optimal removal of NPs under different mass concentrations (1, 2.5 and 10 mg/L) in both systems was found at an FRS of 200 rpm. Moreover, the changes in the removal pattern in both systems were consistent with the variation in the hydraulic conditions. For instance, at an initial concentration of 1 mg/L in the single system of CuO, the effect of the stirring conditions was significantly high. The removal efficiencies of 1, 2.5 and 10 mg/L of nano-CuO using PAFC were found at an FRS of 200 and 350 rpm in the ranges of 69.10% and 28.01%, 95.37% and 38.80% and 95.39% and 98.12%, respectively. Moreover, the removal rates of 1, 2.5 and 10 mg/L of nano-CuO by PAFC/PAM at an FRS of 200 and 350 rpm were found in the ranges of 87.23% and 28.98%, 96.54% and 42.01% and 97.45% and 93.70%, respectively. The variation in stirring conditions significantly affected the removal rates of NPs at lower initial concentrations of 1 and 2.5 mg/L of CuO compared with relatively higher concentrations of 10 mg/L. The addition of MC enhanced the removal rates of nano-CuO and the suspension turbidity under similar conditions. For instance, in the MC suspension system, the removal efficiency of nano-CuO (10 mg/L) using PAFC/PAM at an FRS of 200 and 350 rpm was found as 98.54% and 92.10%, respectively. 

Hydraulic parameters, specifically the agitation time and speed, are major parameters in the formation of stable flocs. Rapid agitation is required for the homogeneous dispersion of the coagulant and flocculant in the sample, while a slower speed is used to form stable flocs. The velocity gradient (G) is another critical parameter influencing the C/F/S performance. Furthermore, mixing speeds also have a direct effect on the formation and stabilization of flocs. During the rapid mixing phase, the rotation speed around 100 rpm was too low and produced a small G value in the solution. Hence, in such systems, the low removal efficiency of nano-CuO was attributed to insignificant floc formation. Furthermore, fast speeds of about 350 rpm produced a higher G value, which was not conducive to floc formation, thereby reducing the removal efficiency of nano-CuO. The optimum G value leads to compact agglomerates with increased fractal dimension and flocs size in the suspension becoming more even [36]. The significant effect of the stirring speed was observed at low concentrations of nano-CuO (1 and 2.5 mg/L) as shown in Figure 4A,B. Such an observation might be related to the fact that the higher agitation rate leads to the formation of small aggregates during the C/F/S process. Further, the tiny and unstable flocs broke down resulting in poor removal efficiency of nano-CuO in a low mass concentration of NPs. Relatively large and stable flocs were formed during the C/F/S process at an initial mass concentration of 10 mg/L, with higher resistance to variations in hydraulic conditions. In the case of PAM alone, the high mixing speed during the C/F/S process resulted in the poor adsorption bridging effect of long-chain polymer [18,37]. Thus, the removal performance of nano-CuO and turbidity were significantly reduced in such a system. 

### 3.5. Response Surface Methodology 

Response surface methodology (RSM) based on the Box–Behnken model (BBD) design principle was used to further understand the removal behavior of nano-CuO (10 mg/L) in MC suspensions. Three independent factors, i.e., PAFC, PAM dosage and stirring conditions, were selected at three levels and the experimental design was set to simulate different C/F/S conditions as shown in Table 1. The removal rate of nano-CuO was taken as the response value and a regression model was generated, as presented in Table 2.

**Figure 5:**
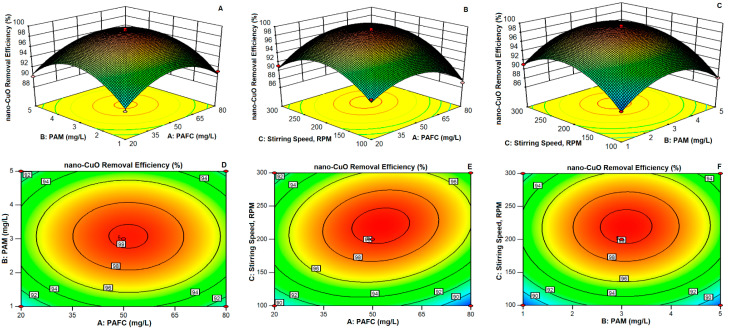
3D surface plots and corresponding contour plots showing the removal rate of nano-CuO under the influences of (**A–D**) PAFC dosage and PAM dosage, (**B**–**E**) PAFC dosage and stirring speed and (**C**–**F**) PAM dosage and stirring speed.

The slope of the response surface plot shows the effect of different parameters on the removal efficiency of nano-CuO as illustrated in Figure 5. The higher slope value indicates the greater influence, whereas the shape of the contour map reflects the effect of the interaction of other parameters on the removal performance. The effect of linear parameters, i.e., hydraulic conditions and PAFC dosage, and all quadratic parameters on the removal efficiency of nano-CuO was found significant. However, the influence of different interactions was found insignificant (Figure 5A–C). At constant PAFC dosage, the removal efficiency of nano-CuO improved and then decreased with the increased PAM dosage (Figure 5A). Likewise, at constant PAM dosage, the increase in PAFC dosage initially increased the removal of nano-CuO and then decreased the overall C/F/S performance (Figure 5B). 

The optimum influencing ranges of PAFC and PAM dosages on the removal of nano-CuO were found to be 37–65 mg/L and 2.20–4.00 mg/L, respectively. However, the model shows no significant effect of the interaction between PAFC and PAM dosages on the removal rate of the pollutant (Table 3). The optimal range of PAFC and stirring speed were found to be 37–65 mg/L and 170–270 rpm, respectively (Figure 5B). The stirring speed had a substantial influence on the removal efficiency of nano-CuO (Table 3). As indicated in Figure 5C, when the PAFC dosage was kept constant, the influence of other parameters on the elimination of nano-CuO initially increased and then decreased. The optimum ranges for the PAM dosage and stirring speed were observed as 2.20–4.00 mg/L and 170–270 rpm, respectively. The regression analysis was performed to further understand the influence of each factor. The regression equation between PAFC, PAM dosage, stirring speed and nano-CuO removal rate is presented in Equation (1).

(1) $$\begin{array}{ll} nano-CuO\;removal & Efficiency\\ & =98.13+0.36A+0.36B+1.83C-0.013AB+1.36AC\\ & +0.22BC-3.90A^{2}-4.55B^{2}-4.72C^{2} \end{array}$$

The detail of the analysis of variance is shown in Table 3. The mathematical model was found significant (F = 80.51, *p* < 0.0001). Moreover, the individual effects of stirring conditions and PAFC dosage were found significant. In contrast, the interaction influence of these parameters was found insignificant. These observations are in good agreement with the results of the response surface analysis. The adjusted coefficient of determination Radj^2^ was found to be 0.9781. Moreover, the obtained experimental results show a good correlation with the estimated value. In general, the model might be utilized to optimize the analysis and estimate the removal of nano-CuO by PAFC/PAM during the C/F/S process.

### 3.6. Model Validation and Monitoring of Floc

The regression equation was solved to find the optimum C/F/S condition for removal of nano-CuO. The obtained settings for NPs’ removal were as follows: PAFC (~52.48 mg/L), PAM (~3.09 mg/L), and stirring conditions of 2 min FRS (220.76 rpm) and 10 min of slow rotation (80 rpm). The calculated value of nano-CuO removal under these environmental conditions was around 99.34%. The precision of the model was further verified by performing the three different sets of the experiment under the obtained model conditions. The results of three identical sets of trials presented that the treatment efficacy of nano-CuO was 97.95%, 98.76% and 99.07%. The calculated relative error among the obtained and estimated value was found to be 0.75%, which shows that the model can better reflect the optimal removal conditions for nano-CuO.

**Figure 6:**
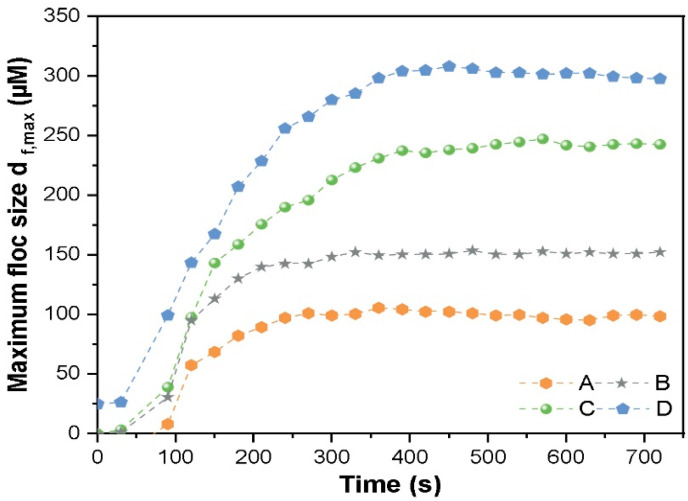
Size of composite floc of nano-CuO (2.5 mg/L) under various conditions at (**A**) 50 mg/L PAFC, (**B**) 50 mg/L PAFC + 20 mg/L MC, (**C**) 50 mg/L PAFC with 3 mg/L PAM and (**D**) 50 mg/L PAFC + 3 mg/L PAM and 20 mg/L MC.

Figure 6 shows the monitoring of floc formation after the flocculation process with various conditions of PAFC, PAM, PAFC/PAM, MC and (2.5 mg/L) of nano-CuO. As demonstrated in Figure 6, the flocs formed under the condition of 50 mg/L PAFC + 3 mg/L PAM and 20 mg/L MC were larger in size as compared to those found at 50 mg/L PAFC + 3 mg/L PAM in pure water. The measured particle size (dia:0.5) of the flocs formed by 50 mg/L PAFC was around 95 µm in pure water, moreover, flocs obtained with 50 mg/L PAFC + 20 mg/L MC was around 145 µm. Thus, the addition of MC enhanced the particle size and density of flocs during flocculation. In contrast, the size of the floc formed by the combination of 50 mg/L PAFC and 3 mg/L PAM was found above 250 µm. The particle size distribution measurement of the flocs after flocculation experiments further revealed that the addition of PAM helped to enhance the floc size above 85%. The excess dosage of PAFC resulted in higher hydrolysis of Al(III) and Fe(III) in coagulation thereby incorporating cations to neutralize the negative surface potential of nano-CuO. However, excess cations in the solution resulted in the formation of tiny flocs with low compactness [38]. The addition of PAM neutralized the negative charge of NPs’ surface thereby destabilizing the suspended NPs in the solution. Moreover, in the presence of PAM, the adsorption bridging occurred during the C/F/S process, which helped PAFC to form large flocs with an enhanced degree of compactness.

### 3.7. Characteristics of Composite Flocs 

**Figure 7:**
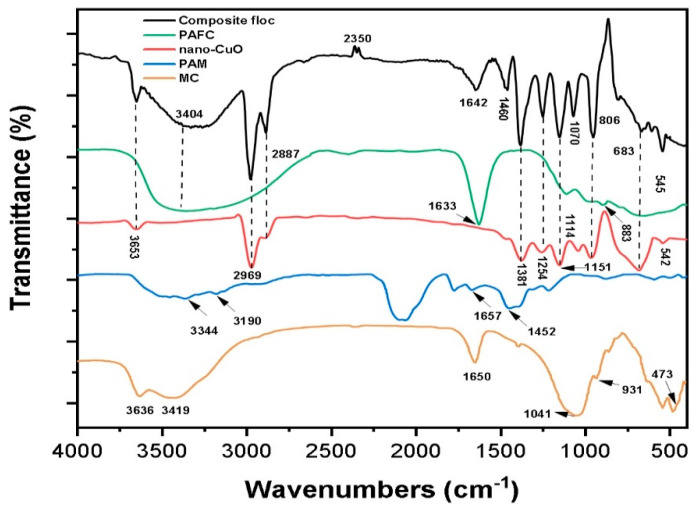
FT-IR spectrum of pristine chemicals, i.e., MC, PAM, nano-CuO, PAFC and composite flocs obtained at optimum C/F/S conditions.

The results of the FTIR analysis of pristine chemicals including MC, PAM, PAFC, nano-CuO and composite flocs obtained after the C/F/S experiment were used to illustrate the probable removal mechanisms, as demonstrated in Figure 7. The observed broad bands at 3300–3400 cm^−1^ due to the stretching vibration of the (^−^OH) functional group linked to aluminum or iron (Al/Fe–OH) [39]. The spectrum peaks observed around 2972, 2887, 1653 and 1460 cm^−1^ were associated with asymmetric and symmetric stretching vibrations of C-H, CH_2_, C=O and acylamino groups, respectively [40]. The absorption bands at 1381 cm^−1^ attributed to the symmetric stretching vibration of CH_3_, COO^−^,while the band appearing at 1254 cm^−1^ corresponded to the C–O anti-symmetric stretching [41]. In addition, few peaks were found at 1257, 1151 and 934 cm^−1^ owing to the stretching of C–OH (phenolic), C–O and carboxylic acid groups [42]. However, the peaks at 883 and 542 cm^−1^ were ascribed to the bending vibration of the Fe–OH– Fe and Cu-O bond stretching, respectively.

In addition, the IR spectra of composite floc obtained by the PAFC/PAM (MC) system showed a broader absorption peak with higher intensity at 3653 cm^−1^ than that of nano-CuO, implying some inner hydroxyl in MC bonded to the Cu-O group of nano metals [43]. The observed peak between 3250 cm^−1^ to 3500 cm^−1^ with strong intensity at 3404 cm^−1^ was larger than that of PAFC, indicating the formation of moderately strong H-bonding between inner surface hydroxyls of the MC and metals ions during the C/F/S process [44,45]. The absorption peak at 1633–1657 cm^−1^ was due to the bending vibration of H–O–H. The peak in the composite floc decreased with the significant shift, indicating that less (OH) groups were combined with Al/Fe ions to form complexes with free Cu ions [46]. The higher intensity of peak at 1460 cm^−1^ and 1254 cm^−1^ attributed to C=O and acylamino groups further clarified the complex coordination with Cu ions in the adsorption process [47]. The bands at 1114 cm^−1^ shifted to a lower frequency at 1070 cm^−1^ with enhanced intensity owing to the stretching vibration of Fe–O/Cu–O. In addition, the peak observed at 683 cm^−1^ was found, suggesting the bending vibration of Fe–O–Cu/Fe–OH–Cu [48]. The enrichment of hydroxyl (OH) bridging in PAFC/PAM (MC) suspension formed the compact structure flocs. Moreover, the flocs formed would increase adsorption and sweep coagulation. This was supported by the findings of floc monitoring, where the increase in floc size was observed in the heterogenous system. The substantial shifts and increase in the intensity of some bands in the obtained composite flocs also well supported the complexations of metal ions and PAFC/PAM in the MC environment. Consequently, it might be concluded from the improved NPs removal rate and IR spectra, that the key mechanism for nano-CuO removal may be the compound effect of charge neutralization, complexation and adsorption.

## 4. Conclusions

In this research, we explored the influence of coagulant dose, pH, settling time and stirring speed on CuO removal during the C/F/S process. The removal efficiency of nano-CuO and turbidity in both systems were enhanced by increasing the initial mass concentration of NPs. Moreover, the presence of MC improved the removal efficiency of CuO. The addition of both PAFC and PAM enhanced the compression and stability of flocs. The surface potential of flocs was greatly influenced in the acidic environment. In addition, the PAFC/PAM significantly increased removal efficiency and reduced the settling time in the MC containing suspension. The variation in initial mass concertation and the stirring speed affected the floc formation and removal performance of nano-CuO. The BBD and RSM techniques were applied to determine optimal C/F/S process conditions for maximizing CuO removal from water. The obtained model responses suggested the optimum C/F/S conditions as PAFC (52.48 mg/L), PAM (3.09 mg/L), and mixing at 2 min of fast rotation (220.76 rpm) and 10 min of slow rotation (80 rpm). Furthermore, the validity of the model was accessed under different environmental conditions. The FT-IR analysis of composite flocs revealed that primary mechanisms including charge neutralization, complexation and adsorption may be involved in the removal of nano-CuO by the C/F/S process. In general, the findings provide insight into enhanced flocculation and coagulation of CuO in drinking water containing clay particles.

## Figures and Tables

**Table 1:** Type, parameters and corresponding coded levels for RSM.

Type	Variables	Coded Level of Variables		
		−1	0	1
		Corresponding Operation Values		
A	PAFC (mg/L)	20	50	80
B	PAM (mg/L)	1	3	5
C	Stirring Speed (RPM)	100	200	350

**Table 2:** Test design and response values for RSM.

Experiment	A	B	C	Nano-CuO Removal (%)
1	0	1	1	92.26
2	−1	1	0	90.15
3	−1	0	1	91.15
4	0	0	0	98.82
5	0	1	−1	88.12
6	0	−1	−1	87.87
7	0	0	0	98.67
8	0	0	0	99.15
9	1	1	0	91.95
10	−1	0	−1	90.23
11	0	0	0	99.48
12	0	0	0	99.51
13	1	0	−1	87.14
14	1	0	1	93.49
15	−1	−1	0	89.37
16	1	−1	0	91.22
17	0	−1	1	91.14

**Table 3:** Analysis of variance.

Source	Sum of Squares	Df	Mean Square	F Value	*p*-Value Prob > F	
Model	310.5	9	34.5	80.51	<0.0001	Significant
A-(PAFC)	1.05	1	1.05	2.45	0.1613	Not significant
B-(PAM)	1.04	1	1.04	2.42	0.1638	Not significant
C-(Stirring Speed)	26.94	1	26.94	62.86	<0.0001	Significant
AB	6.250 ×10^−4^	1	6.25 × 10^−4^	1.458 × 10^−3^	0.9706	Not significant
AC	7.37	1	7.37	17.2	0.0043	Significant
BC	0.19	1	0.19	0.44	0.5276	Not significant
A2	64.02	1	64.02	149.38	<0.0001	Significant
B2	87.33	1	87.33	203.79	<0.0001	Significant
C2	93.97	1	93.97	219.28	<0.0001	Significant
Residual	3	7	0.43			
Lack of Fit	2.42	3	0.81	5.62	0.0643	Not significant
Pure Error	0.57	4	0.14			
Cor Total	313.5	16				

## Data Availability

Not applicable.

## Supplementary Materials

The following are available online at https://www.mdpi.com/article/10.3390/nano11102753/s1, Figure S1: Spectra of pristine nano-CuO, MC and the mixture of nano-CuO + MC.
