# Supplementary material for: Synergetic Effect of Organic Flocculant and Montmorillonite Clay on the Removal of Nano-CuO by Coagulation-Flocculation-Sedimentation Process
Source: Nanomaterials (Basel). 2021 Oct 17;11(10):2753. doi: 10.3390/nano11102753 (PMC8537118; doi:10.3390/nano11102753)
Supplement: Supplementary file 1 [file nanomaterials-11-02753-s001.zip › nanomaterials-1414796-supplementary.pdf]

nanomaterials

Article

Synergetic Effect of Organic Flocculant and Montmorillonite Clay on the Removal of nano-CuO by Coagulation-Flocculation-Sedimentation Process

Rizwan Khan [1], Muhammad Ali Inam [2], Kang Hoon Lee [3,*], Abdul Sami Channa [1], Mukhtiar Ali Mallah [1], Young Min Wie [4] and Mahmood Nabi Abbasi [1]

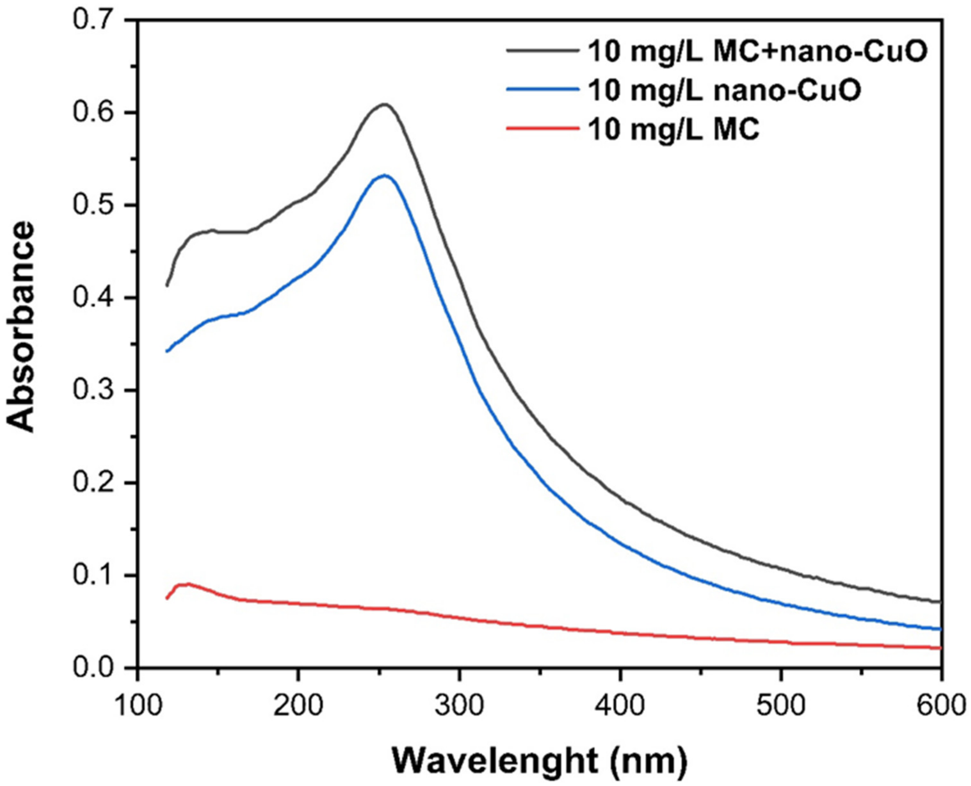

Figure S1. Spectra of pristine nano-CuO, MC and the mixture of nano-CuO + MC.
